# 4311 Inflammatory biomarkers of cognitive dysfunction in pediatric obesity: associations between Executive Function, C-Reactive Protein, Interleukin 6, and Tumor Necrosis Factor alpha

**DOI:** 10.1017/cts.2020.302

**Published:** 2020-07-29

**Authors:** Kathryn Prendergast, Caroline Keller, Shima Dowla, Marissa Gowey

**Affiliations:** 1 University of Alabama at Birmingham

## Abstract

OBJECTIVES/GOALS: Executive function (EF) deficits lead to poorer adherence and weight loss in obesity treatment. Conversely, untreated obesity leads to both EF impairments and risk of chronic disease. EF deficits in children with obesity have begun to be associated with biomarkers of chronic disease risk such as glucose and cortisol. Elevated pro-inflammatory biomarkers, such as C-Reactive Protein (CRP), Interleukin 6 (IL-6), and Tumor Necrosis Factor a (TNFa), indicate risk for obesity-associated chronic disease and may represent another candidate biomarker of EF deficits in pediatric obesity. These inflammatory markers tend to be elevated in obesity but have yet to be examined in association with EF in pediatric obesity. This represents an opportunity to identify potential biomarkers of EF which may serve as novel treatment targets that could improve outcomes and chronic disease prevention. Thus, the present study aims to explore associations between EF and blood serum CRP, IL-6, and TNFa. METHODS/STUDY POPULATION: Treatment-seeking children aged 8-12 years with BMI>95^th^ percentile were recruited from a pediatric weight management clinic and attended baseline assessments for a family-based behavioral intervention program for pediatric obesity. Demographics and medical history were assessed via parent questionnaire. Height and weight were measured by research staff and converted to zBMI. Child performance-based EF was assessed via the NIH Toolbox Cognitive Battery iPad application. Blood draws, assays, and Dual-energy X-ray Absorptiometry (DXA; measuring adiposity) were conducted by trained research personnel. Pearson’s correlations were conducted to explore associations between EF (NIH Toolbox Cognitive Battery, fully corrected T-scores) and CRP, IL-6, and TNFa. RESULTS/ANTICIPATED RESULTS: Children (n = 12) were primarily female (76.5%) and African American (64.7%). Correlation coefficients between inflammatory markers and EF were highly variable (r = −0.39 to 0.52). CRP showed small, negative associations with Cognitive Flexibility (r = −0.39) and Inhibitory Control (r = −0.34). IL-6 also demonstrated a small, negative association with Inhibitory Control (r = −0.37). TNFa was positively and moderately associated with working memory (r = 0.53). Remaining associations were weak (−0.3<r<0.3). DISCUSSION/SIGNIFICANCE OF IMPACT: Signals of higher inflammation, measured via CRP and IL-6, in association with EF deficits were identified in a small sample of children with obesity, as hypothesized. However, signals in the opposite direction were identified as well, measured via TNFa. CRP and IL-6 may represent candidate biomarkers of executive dysfunction in obesity that warrant further domain and biomarker-specific research, with potential long-term implications for improving pediatric obesity treatment.

